# Beyond tractography in brain connectivity mapping with dMRI morphometry and functional networks

**DOI:** 10.1007/s00429-025-03016-1

**Published:** 2025-09-27

**Authors:** Jui-To Wang, Ching-Po Lin, Huei-Min Liu, Carlo Pierpaoli, Chun-Yi Zac Lo

**Affiliations:** 1 Institute of Brain Science, National Yang Ming Chiao Tung University, Taipei, Taiwan; 2 Department of Neurosurgery, Taipei Veterans General Hospital, Taipei, Taiwan; 3 Institute of Neuroscience, National Yang Ming Chiao Tung University, Taipei, Taiwan; 4 Department of Education and Research, Taipei City Hospital, Taipei, Taiwan; 5 Laboratory on Quantitative Medical Imaging, National Institute of Biomedical Imaging and Bioengineering, Bethesda, MD USA; 6 Institute of Intelligent Bioelectrical Engineering, National Yang Ming Chiao Tung University, Hsinchu, Taiwan

**Keywords:** Microstructure, White matter, Functional MRI, Functional correlation tensor, Structure-function coupling

## Abstract

Traditional brain connectivity studies have focused mainly on structural connectivity, often relying on tractography with diffusion MRI (dMRI) to reconstruct white matter pathways. In parallel, studies of functional connectivity have examined correlations in brain activity using fMRI. However, emerging methodologies are advancing our understanding of brain networks. Here we explore advanced connectivity approaches beyond conventional tractography, focusing on dMRI morphometry and the integration of structural and functional connectivity analysis. dMRI morphometry enables quantitative assessment of white matter pathway volumes through statistical comparison with normative populations, while functional connectivity reveals network organization that is not restricted to direct anatomical connections. More recently, approaches that combine diffusion tensor imaging (DTI) with functional correlation tensor (FCT) analysis have been introduced, and these complementary methods provide new perspectives into brain structure–function relationships. Together, such approaches have important implications for neurodevelopmental and neurological disorders as well as brain plasticity. The integration of these methods with artificial intelligence techniques have the potential to support both basic neuroscience research and clinical applications.

## Introduction

This article originates from the ‘Did-You-Know’ sessions held at the TractAnat retreat, where concise insights into emerging topics in brain connectivity were shared. In this contribution, we focus on methods extending beyond tractography - including dMRI morphometry and the structure-function integration - reflecting the themes discussed during the session. This paper forms part of a series of DYK write-ups stemming from the retreat.

The human brain represents one of the most complex networks in nature, with its intricate web of connections forming the foundation for cognition, behavior, and consciousness. Traditionally, the study of brain connectivity has relied heavily on tractography, a method that uses diffusion MRI (dMRI) to reconstruct white matter fiber pathways and estimate connectivity between brain regions through connectome analysis. Tractography, based on streamline reconstructions, reflects structural connectivity by estimating pathways between regions, often using streamline counts as a proxy for connection strength (Maier-Hein et al. 2017). In contrast, dMRI morphometry quantifies geometrical and microstructural metrics of white matter pathways (Sadeghi et al. 2018). Recent methodologies, including the newer dMRI morphometry and the well-established functional connectivity analysis, provide complementary perspectives for studying brain networks, offering deeper insights that extend beyond the limitations of traditional tractography.

## dMRI morphometry: principles and applications

### Technical fundamentals

The emergence of dMRI morphometry represents a methodological shift from conventional tractography methods. This approach operates on fundamentally different principles than traditional fiber tracking. Rather than attempting to reconstruct individual white matter pathways, dMRI morphometry focuses on statistical quantification of white matter microstructure and pathway volumes (Alexander et al. 2019). Traditional scalar analyses of diffusion MRI, such as comparing fractional anisotropy (FA) or mean diffusivity (MD) across regions of interest, provide indirect measures of white matter microstructural integrity but are not morphometric in the strict sense. In contrast, diffusion morphometry approaches aim to capture the geometry and spatial characteristics of white matter pathways. Early methods include voxelwise frameworks such as Tract-Based Spatial Statistics (TBSS), which evaluates diffusion metrics along a common white matter skeleton (Smith et al. 2006), and tract-specific volumetric analyses that quantify the spatial extent of predefined bundles.

More recently, diffusion tensor–based morphometry (DTBM) has been developed to incorporate both scalar and directional information from the diffusion tensor into the registration process. In DTBM, individual diffusion tensor images are nonlinearly registered to a population template using tensor-derived similarity metrics, which produces deformation fields describing how each subject’s anatomy is aligned to the template. The Jacobian determinants of these deformation fields are then analyzed to estimate local expansions or contractions in white matter regions. By relying on tensor-driven registration rather than scalar maps alone, DTBM allows the study of regional variations in white matter morphology (Sadeghi et al. 2018). Unlike tractography, which reconstructs streamlines to infer connectivity patterns, DTBM characterizes the geometry and local volumetric properties of white matter pathways, and results can be compared across individuals and groups (Sadeghi et al. 2020) .

### Clinical applications and broader impact

**Fig. 1:**
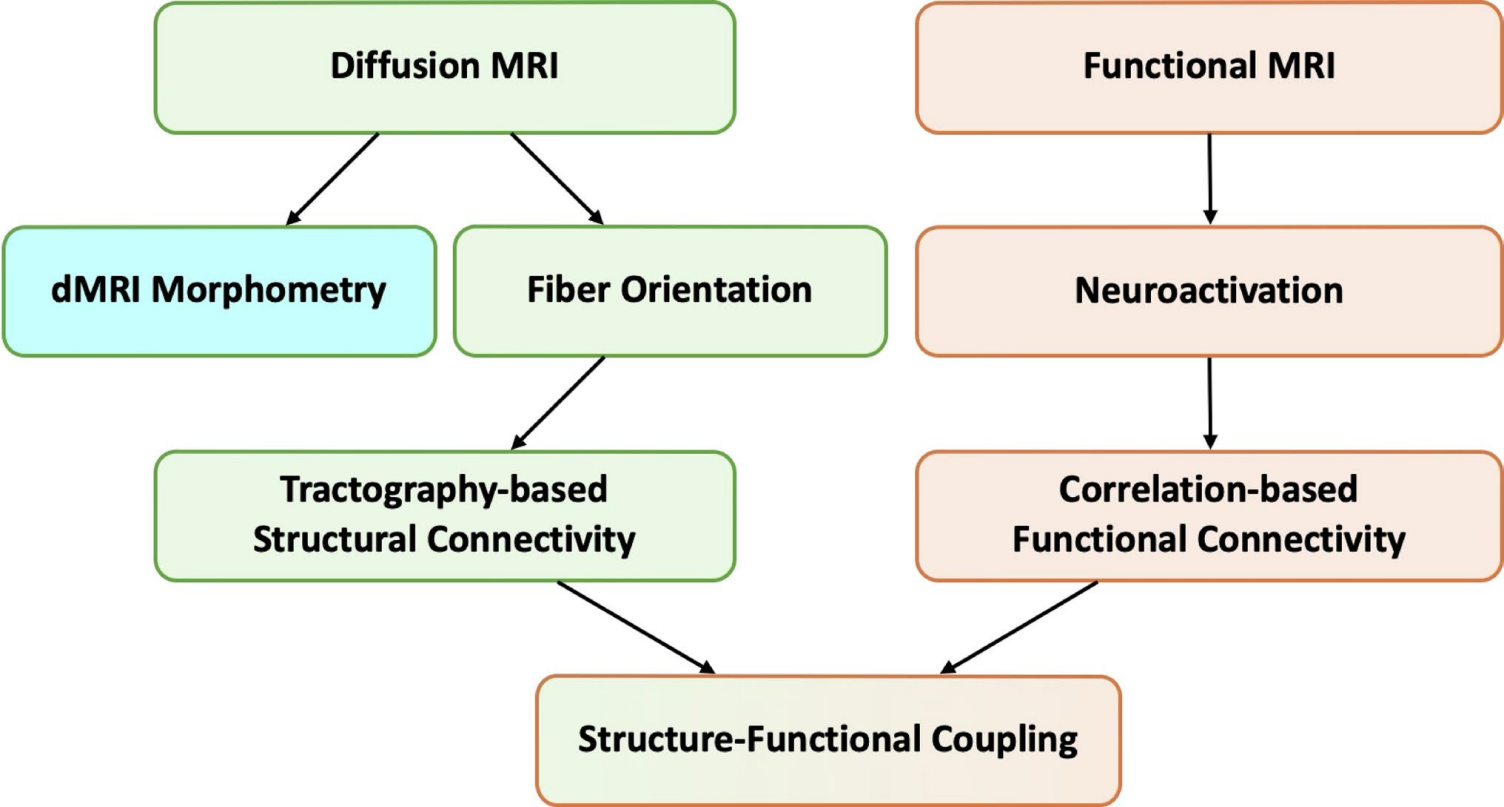
Conceptual schematic of modalities, methods, connectivity domains, and integration. The schematic illustrates a flow from imaging modalities to integration. At the top, diffusion MRI (dMRI) and functional MRI (fMRI) serve as complementary inputs. From dMRI, two analyses are highlighted: morphometry, which employs deformation fields to enhance sensitivity for detecting morphological abnormalities in specific white matter pathways, and fiber orientation, which supports tractography. From fMRI, neuroactivation measures contribute to functional analyses. These methods converge into two connectivity domains: tractography-based structural connectivity on the dMRI side, and correlation-based functional connectivity on the fMRI side. Finally, both domains merge at the bottom into structure–function coupling, providing a conceptual framework to relate microstructural features with large-scale functional networks and to inform both neuroscience research and potential clinical applications

Recent applications of dMRI morphometry have yielded useful insights across a spectrum of neurological conditions. A Moebius syndrome example shown in Fig. 1 illustrates this approach, which has demonstrated significant utility across neurodevelopmental and neurological disorders ranging from rare to common conditions (Sadeghi et al. 2020). In autism spectrum disorders, morphometric analysis has revealed consistent patterns of altered white matter organization in language and social cognition networks (Travers et al. 2012). Studies of attention deficit hyperactivity disorder (ADHD) have identified specific reductions in white matter pathway volumes connecting frontal and striatal regions, providing neurobiological markers that correlate with symptom severity(Nagel et al. 2011) .

The technique has also proven valuable in white matter disorders such as multiple sclerosis, where morphometric measures can detect subtle changes in lesion burden and normal-appearing white matter before they become visible on conventional MRI (Vrenken et al. 2006). In aging research, longitudinal morphometric studies have characterized the patterns and rates of white matter deterioration, contributing to our understanding of healthy aging versus pathological processes like Alzheimer’s disease (Salat et al. 2005) .

## Functional connectivity beyond structural constraints

### Methodological principles

Functional connectivity analysis represents a complementary approach that examines the temporal correlations between brain regions’ activity patterns, typically measured through functional MRI (fMRI) blood-oxygen-level-dependent (BOLD) signals (Biswal et al. 1995; Shahhosseini and Miranda 2022). The fundamental principle involves computing statistical dependencies between time series of brain activity across different regions, regardless of direct anatomical connections. This analysis can be performed using various approaches, including seed-based connectivity (examining correlations between a predefined region and the rest of the brain), independent component analysis (identifying spatially distributed networks with coherent temporal dynamics), and graph-theoretical approaches that characterize network topology properties(Beckmann and Smith 2004; Bassett and Bullmore 2009) .

Resting-state functional connectivity has revealed intrinsic brain networks that maintain consistent activity patterns even in the absence of specific tasks (Fox et al. 2005). These networks, including the default mode network, salience network, and executive control networks, demonstrate remarkable consistency across individuals while also showing meaningful variations related to cognitive abilities, personality traits, and clinical conditions (Lynch et al. 2024; Zhang et al. 2024) .

### Structure-function relationships

The relationship between structural and functional connectivity represents an important area in brain mapping research. Functional connectivity patterns, as revealed through fMRI techniques, often extend far beyond the constraints of direct anatomical connections identified through tractography (Honey et al. 2009). This phenomenon demonstrates the brain’s capacity for dynamic organization and adaptation through polysynaptic pathways and indirect network effects.

**Fig. 2:**
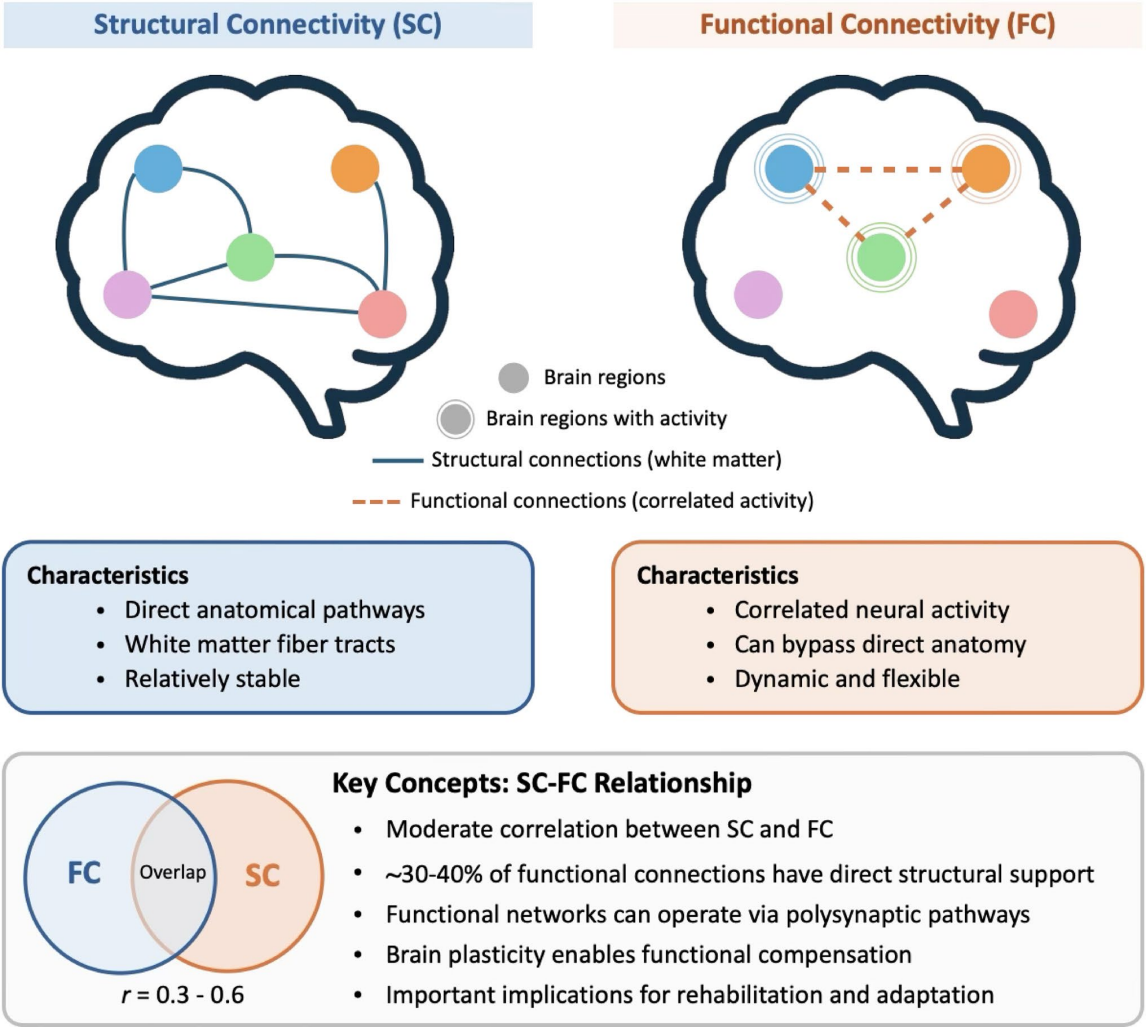
Structure-function connectivity relationships and clinical implications. Brain networks can be characterized through both structural and functional approaches that provide complementary information. Structural connectivity (SC) maps direct white matter pathways, while functional connectivity (FC) identifies temporal correlations in neural activity. The moderate correlation between SC and FC (0.3–0.6) reveals that functional networks can operate beyond anatomical constraints, enabling brain plasticity and compensatory mechanisms. This dissociation has important implications for understanding healthy brain function, injury recovery, and neurodevelopmental disorders

Quantitative studies have demonstrated that structural and functional connectivity matrices show only moderate correlations, with overlap coefficients typically ranging from 0.3 to 0.6 depending on the analysis methods and brain regions examined (Schulz et al. 2008). This dissociation suggests that functional networks operate with considerable independence from their direct structural foundations, relying on complex multi-step pathways and network-level interactions (Fig. 2). Recent advances have enabled more direct assessment of structure-function coupling within white matter itself, moving beyond traditional approaches that separately measure structural properties in white matter and functional connectivity in gray matter(Zhao et al. 2023). Novel methodologies such as functional correlation tensor (FCT) analysis provides new perspectives on how functional signals align with white matter pathways (Ding et al. 2016). When combined FCT with diffusion tensor measurements of local fiber orientation, these approaches reveal that structure-function coupling varies significantly across different brain regions and can be disrupted in neurological and psychiatric conditions. For instance, studies in schizophrenia have demonstrated widespread decreases in structure-function coupling within white matter regions, particularly affecting the corticospinal tract and superior longitudinal fasciculus, which correlate with clinical symptoms and disease duration (Zhao et al. 2023).

## Integration and clinical implications

### Multimodal approaches

The complementary nature of these different approaches to studying brain connectivity has created opportunities for research in both basic neuroscience and clinical applications. By integrating diffusion model–derived structural connectivity and functional connectivity (e.g., FCT), researchers can develop more comprehensive models of brain organization (Calhoun and Sui 2016). This multimodal approach provides insights into how structural alterations relate to functional reorganization, particularly relevant in studies of brain plasticity following injury or in neurodevelopmental conditions.

Recent studies have demonstrated that individuals with similar structural connectivity profiles may exhibit markedly different functional connectivity patterns, suggesting that functional networks can compensate for structural limitations through alternative routing mechanisms. This flexibility in functional organization helps explain how individuals can maintain cognitive function despite structural brain changes due to aging, injury, or disease (Reuter-Lorenz and Park 2010) .

### Clinical translation

The clinical implications of these advanced connectivity approaches are particularly significant. In neurodevelopmental disorders, the ability to quantify and compare white matter pathway volumes against normative data can help identify subtle structural differences that contribute to cognitive or behavioral symptoms. Functional connectivity analysis can reveal aberrant network patterns that may not be apparent from structural imaging alone.

Understanding how functional networks can operate beyond structural constraints informs therapeutic strategies aimed at promoting neural plasticity and functional recovery after brain injury. For example, rehabilitation protocols can be designed to strengthen alternative functional pathways when primary structural connections are compromised. Recent work has also demonstrated that structure-function coupling measurements can serve as sensitive biomarkers for psychiatric conditions, with the potential to track treatment responses and disease progression (Zhao et al. 2023). This represents an important step from traditional approaches that examine structural and functional properties in isolation, providing a more comprehensive understanding of how brain networks support cognition and behavior.

### Future directions and technological integration

The integration of artificial intelligence and machine learning techniques with these advanced connectivity approaches represents a developing area with multiple applications. Deep learning algorithms can identify complex, non-linear patterns in multimodal connectivity data that traditional statistical methods might miss (Vieira et al. 2017). Specifically, convolutional neural networks have been applied to automatically segment white matter pathways from morphometric data, while recurrent neural networks can model the temporal dynamics of functional connectivity patterns.

Machine learning approaches enable the development of predictive models that combine structural and functional connectivity features to classify clinical conditions, predict treatment outcomes, or identify individuals at risk for neurological disorders. Graph neural networks represent an emerging approach for analyzing brain connectivity data, as they can naturally incorporate the network structure of brain connections while learning complex relationships between local and global network properties (Parisot et al. 2018) .

## Conclusion

Diffusion MRI morphometry has emerged as a promising tool to quantify white matter microstructural variations beyond conventional tractography, offering new perspectives on brain organization. At the same time, the integration of diffusion MRI and functional connectivity analyses—particularly the coupling of DTI-based structural connectivity with fMRI-derived functional connectivity (or FCT)—is providing additional insights into how structural and functional networks interact. Together, these complementary approaches are advancing our understanding of brain organization and suggesting potential applications for clinical applications, from early detection of neurological conditions to personalized therapeutic interventions.

As technology continues to advance and analytical capabilities expand through artificial intelligence integration, these approaches will likely play an increasingly important role in both neuroscience research and clinical practice. Future developments may yield more sophisticated tools for understanding the brain’s connectivity patterns, ultimately contributing to better treatments for neurological conditions and a deeper comprehension of human brain function. The evolution from simple tract tracing to comprehensive network analysis represents not just a methodological advance, but a meaningful change in how we study the connected brain.

## Data Availability

No datasets were generated or analysed during the current study.
